# Supplementary material for: Silver Nanoparticle-Anchored Human Hair Kerateine/PEO/PVA Nanofibers for Antibacterial Application and Cell Proliferation
Source: Molecules. 2021 May 8;26(9):2783. doi: 10.3390/molecules26092783 (PMC8125921; doi:10.3390/molecules26092783)
Supplement: Supplementary file 1 [file molecules-26-02783-s001.zip › molecules-1196924-supplementary.pdf]

molecules

MDPI

Supplementary materials

Silver Nanoparticle-Anchored Human Hair Kerateine/PEO/PVA Nanofibers for Antibacterial Application and Cell Proliferation

Jiapeng Tang[1,2], Xiwen Liu[1,2], Yan Ge[3,4]* and Fangfang Wang[5]

1. Department of Physiology and Hypoxic Biomedicine, Institute of Special Environmental Medicine, Nantong University, Nantong 226019, China
2. Co-innovation Center of Neuroregeneration, Nantong University, Nantong 226001, China.
3. School of Textile and Clothing, Nantong University, Nantong 226019, China
4. National & Local Joint Engineering Research Center of Technical Fiber Composites for Safety and Protection, Nantong University, Nantong 226019, China
5 College of Fine Arts and Design, Yangzhou University, Yangzhou 225009, China

* Correspondence: author: Yan Ge, Ph.D (E-mail: geyan@ntu.edu.cn)

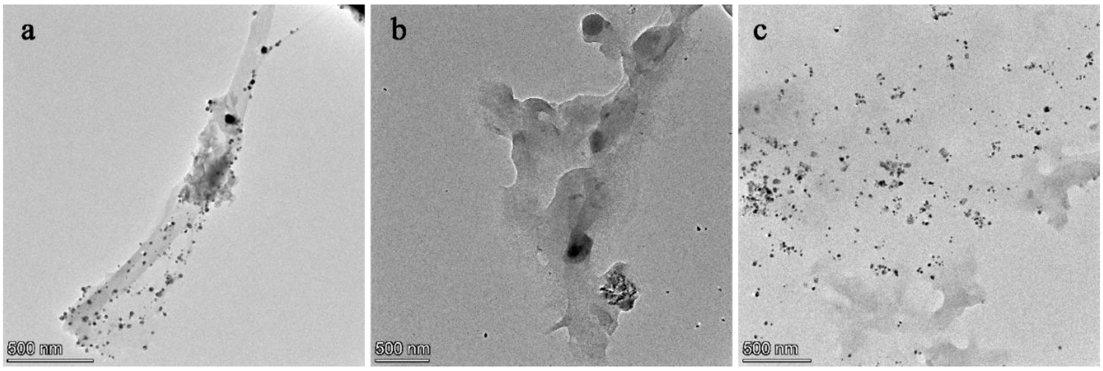

Figure S1. TEM images of AgNP-HHK/PEO/PVA nanofibers treated with (a) 0.005 M, (b) 0.01 M, and (c) 0.02 M of silver nitrate solution.

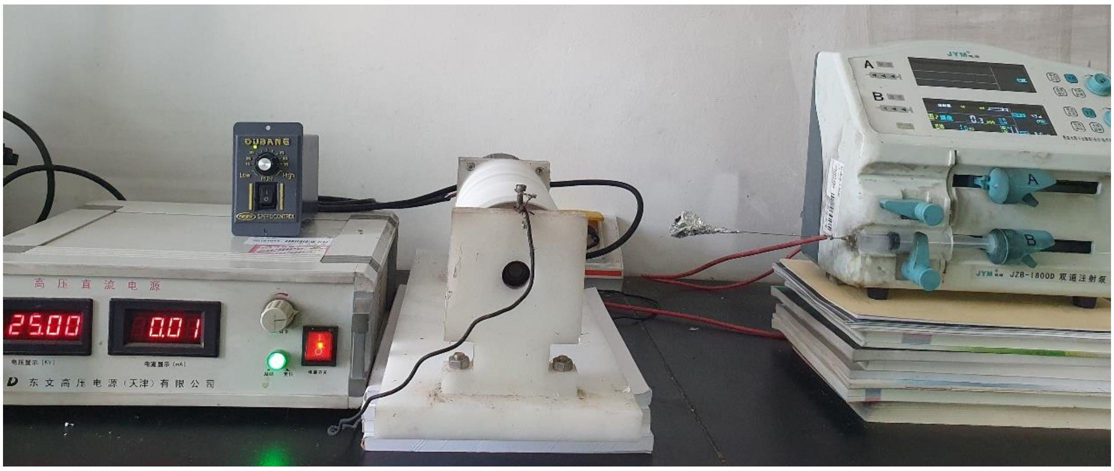

Figure S2. The electrospinning process and equipment.
